# Carbon Dioxide and Nitrogen Infused Compressed Air Foam for Depopulation of Caged Laying Hens

**DOI:** 10.3390/ani8010006

**Published:** 2018-01-03

**Authors:** Shailesh Gurung, Dima White, Gregory Archer, Darrel Styles, Dan Zhao, Yuhua Farnell, James Byrd, Morgan Farnell

**Affiliations:** 1 Department of Poultry Science, Texas A&M AgriLife Research and Extension, College Station, TX 77843, USA; sg45902@tamu.edu (S.G.); dima.white@vikings.berry.edu (D.W.); garcher@tamu.edu (G.A.); dz137@tamu.edu (D.Z.); yfarnell@tamu.edu (Y.F.); 2 US Department of Agriculture, Animal and Plant Health Inspection Service-Veterinary Services, Riverdale Park, MD 20737, USA; darrel.k.styles@aphis.usda.gov; 3 Southern Plains Agricultural Research Center, Agricultural Research Service, US Department of Agriculture, College Station, TX 77845, USA; byrdmen8@yahoo.com

**Keywords:** depopulation, compressed air foam, corticosterone, serotonin, cessation of movement

## Abstract

**Simple Summary:**

Compressed air, detergent, and water make up compressed air foam. Our laboratory has previously reported that compressed air foam may be an effective method for mass depopulation of caged layer hens. Gases, such as carbon dioxide and nitrogen, have also been used for poultry euthanasia and depopulation. The objective of this study was to produce compressed air foam infused with carbon dioxide or nitrogen to compare its efficacy against foam with air and gas inhalation methods (carbon dioxide or nitrogen) for depopulation of caged laying hens. The study showed that a carbon dioxide-air mixture or 100% nitrogen can replace air to make compressed air foam. However, the foam with carbon dioxide had poor foam quality compared to the foam with air or nitrogen. The physiological stress response of hens subjected to foam treatments with and without gas infusion did not differ significantly. Hens exposed to foam with nitrogen died earlier as compared to methods such as foam with air and carbon dioxide. The authors conclude that infusion of nitrogen into compressed air foam results in better foam quality and shortened time to death as compared to the addition of carbon dioxide.

**Abstract:**

Depopulation of infected poultry flocks is a key strategy to control and contain reportable diseases. Water-based foam, carbon dioxide inhalation, and ventilation shutdown are depopulation methods available to the poultry industry. Unfortunately, these methods have limited usage in caged layer hen operations. Personnel safety and welfare of birds are equally important factors to consider during emergency depopulation procedures. We have previously reported that compressed air foam (CAF) is an alternative method for depopulation of caged layer hens. We hypothesized that infusion of gases, such as carbon dioxide (CO_2_) and nitrogen (N_2_), into the CAF would reduce physiological stress and shorten time to cessation of movement. The study had six treatments, namely a negative control, CO_2_ inhalation, N_2_ inhalation, CAF with air (CAF Air), CAF with 50% CO_2_ (CAF CO_2_), and CAF with 100% N_2_ (CAF N_2_). Four spent hens were randomly assigned to one of these treatments on each of the eight replication days. A total of 192 spent hens were used in this study. Serum corticosterone and serotonin levels were measured and compared between treatments. Time to cessation of movement of spent hens was determined using accelerometers. The addition of CO_2_ in CAF significantly reduced the foam quality while the addition of N_2_ did not. The corticosterone and serotonin levels of spent hens subjected to foam (CAF, CAF CO_2_, CAF N_2_) and gas inhalation (CO_2_, N_2_) treatments did not differ significantly. The time to cessation of movement of spent hens in the CAF N_2_ treatment was significantly shorter than CAF and CAF CO_2_ treatments but longer than the gas inhalation treatments. These data suggest that the addition of N_2_ is advantageous in terms of shortening time to death and improved foam quality as compared to the CAF CO_2_ treatment.

## 1. Introduction

The U.S. poultry industry lost 50.4 million birds (layers, turkeys, and backyard flocks) in 15 states during the 2014–2015 highly pathogenic avian influenza (HPAI) outbreak [1]. The overall economic loss was estimated to be $3.3 billion [2]. In addition to HPAI, outbreaks of other reportable diseases, such as exotic Newcastle disease (END), infectious laryngotracheitis, mycoplasmosis, and Marek’s disease, have occurred in the past and pose significant risks to the industry [3]. The 2002–2003 California END outbreak resulted in the loss of 3.16 million birds and cost $180 million in federal money to remediate [4]. Protecting poultry from reportable diseases is still a major challenge facing the industry today [5].

Euthanasia and depopulation methods are used to eliminate animals infected or suspected of infection after confirmation of a reportable disease. The American Veterinary Medical Association (AVMA) defines euthanasia as an act of killing animals in a way that causes no or minimum pain and suffering. Depopulation, on the other hand, refers to an emergency measure to rapidly eliminate animals with as much consideration given to their welfare as possible [6]. These methods are vital for controlling the multiplication and spread of a reportable disease. The Animal and Plant Health Inspection Service (APHIS) depopulation goal during an HPAI outbreak is to kill infected poultry within 24 h of a presumptive diagnosis of a case [7]. The timing of depopulation is important to contain the disease, prevent further cases of infection, eradicate the pathogen, and facilitate business continuity [8]. 

Current poultry depopulation methods can be broadly categorized as gas inhalation and foam-based methods [6,7]. The most commonly used gas for mass depopulation during disease outbreaks is carbon dioxide. Carbon dioxide (CO_2_) has been widely used as a means of euthanizing laboratory animals and stunning broilers, pigs, and turkeys in slaughter plants [9,10,11]. It is an analgesic and anesthetic gas [12] which causes rapid loss of consciousness by decreasing intracellular pH [13]. Chickens exposed to 45–50% CO_2_ die within 2 to 5 min of exposure [14]. Water-based foam has been approved as a means for depopulation of floor reared poultry by the AVMA [15]. Foam is a collection of air filled bubbles produced from a solution of water and foam concentrate (detergents). Benson et al. [16] developed water-based foam as a method of depopulation in response to the Delmarva AI event of 2004. Poultry houses are flooded with the foam which forms a thick blanket around birds. Birds die due to mechanical hypoxia as a result of an obstruction of the respiratory tract [16,17]. The advantages of this method are minimum safety risks, limited human contact with infected birds, no requirement for tight sealing of poultry houses, reduction in dusts and aerosols, and rapid depopulation. Ventilation shutdown was recently implemented as a method of depopulation by the USDA-APHIS to meet the 24 h depopulation goal [7]. During ventilation shutdown the birds in poultry houses are deprived of natural or mechanical ventilation with or without increasing the temperature. The birds ultimately die from hyperthermia [18]. 

However, these methods have limitations and associated risks to use in commercial cage layer farms. The use of CO_2_ is not suitable for all kinds of poultry houses as it requires effective sealing, needs special monitoring and safety equipment, and has safety risks for the personnel involved [16,19]. Chickens demonstrate aversive signs to CO_2_ inhalation [20] as they possess intrapulmonary chemoreceptors for the gas [21,22]. Aspirated and high expansion foams, used for floor-reared poultry, are not suitable for commercial caged layer operations. Caged layer houses present a different challenge for foam depopulation due to high stocking densities (100,000 or more layers per house), mesh cage floors that prevent foam build up, and multi-tier buildings (5–10 tiers of cages) which limit access to foam [23]. It is essential to develop alternative methods to rapidly and humanely depopulate caged layer hens during disease outbreaks. Ventilation shutdown is used only when all other methods are found to be inadequate to contain the spread of a pathogen [24,25]. However, ventilation shutdown presents significant challenges to bird welfare.

A compressed air foam (CAF) system is a widely used firefighting technique which makes use of foam concentrate, water, and compressed air to make a finished foam [26,27,28]. White and colleagues [29] reported that the application of disinfectants such as peroxyacetic acid and glutaraldehyde using a CAF system significantly reduces the aerobic bacterial population in commercial layer houses. A study in our laboratory found that CAF can be used as an alternative method for depopulation of caged layer hens (paper under review). The foam and water solution is mixed inside a mixing chamber with compressed air in a CAF system [26]. The ratio of aqueous foam solution and compressed air can be changed as desired to produce drier or wetter foam [30]. It is important that foam used for depopulation in cage operations (conventional, colony, or enriched colony) has a longer dewatering time and a small bubble size. Such characteristics would allow foam to persist long enough in cages, depriving hens of oxygen and ultimately causing their death from hypoxia [31]. Compressed air foam has a longer drainage time and uniform bubble size compared to aspirated foam [32]. Gases, such as CO_2_ or N_2_, can be used instead of air to make CAF since a CAF unit is a closed system. Benson et al. [16] reported the addition of CO_2_ into the finished CAF using a gas injection nozzle for floor-reared poultry depopulation. However, the concentration of CO_2_ in the foam was 1% or less as reported by Benson and colleagues [16]. In our study, CO_2_ or N_2_ was infused to create an aqueous foam solution in the mixing chamber of the CAF system to make the finished foam. 

We hypothesized that the addition of 40–50% CO_2_ in air or 100% N_2_ to make CAF would reduce physiological stress and shorten the time to the cessation of movement. The aim of the study was to evaluate the efficacy of CAF infused with CO_2_ or N_2_ to depopulate caged layer hens. The specific objectives were to develop CAF with CO_2_ or N_2_, to evaluate physiological responses of laying hens subjected to the treatments, and to determine time to cessation of movement of hens to estimate the time to death.

## 2. Materials and Methods

### 2.1. Test Subjects

A total of 192 Lohman LSL spent hens of at least 90 weeks of age, were obtained from an egg integrator. These hens were housed at a layer barn in the Texas A&M University, Poultry Science Research, Teaching and Extension Center. Layer hens were housed in floor pens with access to the outdoors. These birds were caught by hand and placed in coops for transport to our field laboratory on the day of each experiment. The birds were then removed as needed and placed into our cage system for the treatments. The hens were supplied with clean drinking water and a diet that met or exceeded industry recommendations. These birds were cared for following an approved Institutional Animal Care and Use Committee protocol (IACUC 2016–0221).

### 2.2. Experimental Design

Spent hens were subjected to six treatments. The treatments consisted of a negative control (NEG), 50% CO_2_ in air (CO_2_), 100% N_2_ (N_2_), CAF with air (CAF), CAF with 50% CO_2_ (CAF CO_2_), and CAF with 100% N_2_ (CAF N_2_). Four spent hens were chosen from a communal floor pen and randomly assigned to each treatment. Each treatment was replicated eight times over a period of four months. A total of 192 spent hens were used in the entire study. The birds were placed in a conventional pullet cage of 0.61 m × 0.57 m × 0.38 m dimensions, per section, suspended above a plywood bottom to simulate a manure belt. One section of the cage system was sealed with plastic sheeting and duct tape, which was used for the gas inhalation treatments only. On each replicate day all six treatments were performed one after another. The order of the treatments were CAF CO_2_, CO_2_, N_2_, CAF N_2_, CAF, and NEG for all eight replicates. This same order was followed due to the logistics of the foam/gas production process. Each experiment started at 8:00 a.m. and ended by 10:00 a.m. Hens in the NEG group were placed inside the cage for the same duration as the other treatments before removing them for blood collection.

### 2.3. Foam Production and Application

**Figure 1:**
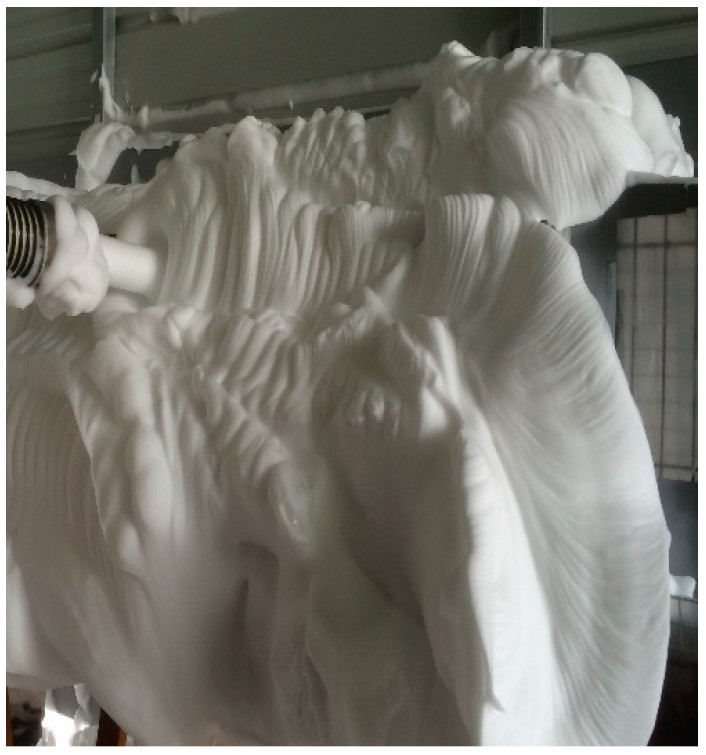
Application of compressed air foam into cages.

**Figure 2:**
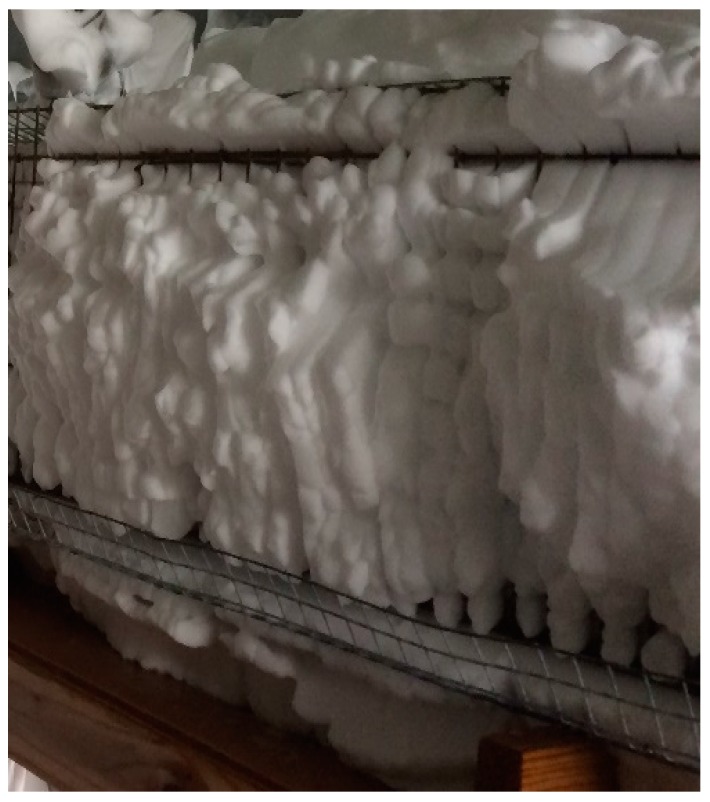
Cages filled with compressed air foam.

The components of a compressed air foam system (CAFS; Rowe CAFS LLC, Hope, AR, USA) were a 1982 L/m (70 cfm) rotary screw air compressor (Vanair Inc., Michigan City, IN, USA), a 29.42 kW (40 HP) gasoline engine (Kohler, Kohler, WI, USA), a 567 L/m (150 gal/m) centrifugal water pump (Hale Products, Inc., Ocala, FL, USA), and a foam proportioning unit (0.1–10%) (FoamPro, Kingston, NY, USA). The foam proportioning unit injected Class A foam concentrate (ICL Performance Products, Rancho Cucamonga, CA, USA) into the water manifold of the CAF unit to make a 3.5% foam water solution. A 1136 L (300 gal) water tank installed on the trailer bed supplied water for producing foam. A separate air manifold supplied compressed gases to the mixing chamber from the air compressor or vaporizer. The three constituents (gas, water, and foam concentrate) were agitated in the mixing chamber of the CAFS unit. Foam of a desired consistency and thickness was produced by adjusting the flow of aqueous foam solution. Foam quality was determined visually by the ability of the foam to properly fill the cage. Figure 1 and Figure 2 illustrate the consistency and thickness of foam produced during the experiment. A 6.4 cm wide and 6 m long suction hose connected to a 3.8 cm CAF system through 15 m of firefighting hose of the same diameter was used to deliver CAF to the spent hens. The foam was applied to the cages for two minutes and allowed to stay in the cages for an additional two minutes. After the end of the four-minute period, foam was washed away and hens were immediately removed from the cages.

### 2.4. CAF Infused with Gases

Liquid CO_2_ and N_2_ tanks delivered respective gases to produce CAF CO_2_ and CAF N_2_ foam. The liquid gases were heated using a 480 volt vaporizer set at 65 °C (Thermax Inc., North Dartmouth, MA, USA) before flowing through mass flow controllers (Alicat, Tucson, AZ, USA). In the CAF CO_2_ treatment, compressed air from the air compressor was first diverted through two consecutive water/oil separators, a desiccant dryer and, finally, a particulate filter before flowing through the mass flow controller. The flow rates of CO_2_ and compressed air were the same, 453 L/m (16 cfm) each, to obtain a gas mixture of equal parts of CO_2_ and air. The gas mixture was then agitated with the foam water solution in the mixing chamber to make CAF CO_2_ foam. The mixing tank was completely emptied each time before another gas was filled in. In the case of CAF infused with N_2_, 100% N_2_ gas was mixed with the foam water solution in a mixing chamber to make CAF N_2_ foam. The flow rate of N_2_ gas was adjusted at 906 L/m (32 cfm). A 25 kVA diesel engine generator (Multiquip Inc., Carson, CA, USA) supplied power necessary for the vaporizer and mass flow controller. An infrared CO_2_ analyzer was used to measure the concentration of the gas in the finished foam (Servomex, Crowborough, UK). 

### 2.5. Gas Inhalation Treatments

Thick polyethylene was fixed to the sides of a cage with duct tape to make an enclosed chamber for the 50% CO_2_ and 100% N_2_ treatments. The gases were introduced into the chamber using the same hoses used for the application of the foam treatments.

### 2.6. Measurement of Expansion Ratio and CO_2_ Concentration

Foam samples were collected in 125 L containers. The foam was allowed to dewater overnight and the aqueous foam solution at the bottom of the container was measured using a graduated cylinder. The same procedure was followed for all three kinds of foam samples, CAF, CAF CO_2_, and CAF N_2_. The expansion ratio was calculated as the ratio of the volume of the finished foam to the volume of aqueous foam solution. 

Foam samples from the CAF CO_2_ treatment were collected in 3.8 L zip-lock bags. These bags were sprayed with a 10% anti-foam solution (Sigma-Aldrich, St. Louis, MO, USA) to allow foam bubbles to rupture releasing CO_2_ for measurement of the gas concentration in the samples. The infrared CO_2_ analyzer was calibrated using 80% CO_2_ calibration gas prior to the measurements. A 20-gauge needle, connected through a delivery hose to the analyzer, was inserted into the top of each sample bag headspace for measurement of the CO_2_ levels. Four samples were measured in each replication. 

### 2.7. Assessment of Stress Hormones

Death was ascertained by observing corneal and pedal reflexes of the spent hens prior to the collection of blood. Blood samples were collected immediately (within two minutes of death) from each individual bird postmortem by severing the femoral artery, except in the NEG group. As birds in the NEG group were alive during the entire treatment period, blood was collected by venipuncture from the jugular vein at the end of the four minute treatment period. The birds in the NEG group were euthanized by cervical dislocation after collection of blood. The blood samples were allowed to clot overnight at 4 °C before being centrifuged at 1000× *g* for 10 min at 4 °C to collect serum. Serum corticosterone (CORT) and serotonin (5-HT) levels were determined using competitive ELISA assay kits ADI-901-097 and ADI-900-175, respectively (Enzo Life Sciences, Farmingdale, NY, USA) according to the manufacturer’s directions. Three spent hens subjected to the CAF treatment and one exposed to CAF CO_2_ treatment had survived. Hens that recovered were immediately euthanized by cervical dislocation. Two blood samples of hens in the negative control and one sample in the CAF treatment group did not yield enough serum. Therefore, out of a total of 192 spent hens used in the study, only 185 serum samples were processed by ELISA. The number of samples used in the CORT assay for each of the six treatments: NEG, CO_2_, N_2_, CAF, CAF CO_2_, and CAF N_2_ were 30, 32, 32, 28, 31, and 32, respectively. In order to assess the 5-HT levels, sixteen serum samples from each treatment group were used except in the CAF CO_2_ treatment (only 15 samples due to availability). Hence, the total number of samples for 5-HT assays was 95. Serum samples were run in duplicates for the CORT and 5-HT assays. The intra-assay and inter-assay variability of the corticosterone assay were 2.25% and 8.3%, respectively. The intra-assay and inter-assay variability of the 5-HT assay were 2.3% and 5.7%, respectively.

### 2.8. Determination of Cessation of Movement

In each of the six treatments, accelerometers (Hobo Pendant G data logger, Onset Computer Corporation, Bourne, MA, USA) were attached to each bird before placing them into a cage [33]. All four birds in each treatment had accelerometers attached to their shanks using nylon wire ties. However, data from spent hens in the NEG group were not used for statistical analysis. Time to cessation of movement (COM) was determined based on the accelerometer readings. The time to COM was calculated as the difference in time from the application of treatment to cessation of convulsive movements as indicated by a flat line in the accelerometer readings. Three spent hens in the CAF treatment and one in the CAF CO_2_ treatment had survived the process. In addition, accelerometers fell off the shank of three hens exposed to CO_2_ treatments and one of the hens subjected to CAF. Therefore, COM data was collected from 152 spent hens.

### 2.9. Statistical Analysis

All data collected on CORT and 5-HT concentrations as well as time to COM from accelerometers were compiled in a spreadsheet (MS-EXCEL, Microsoft, Santa Rosa, CA, USA). The CORT, 5-HT, and time to COM data from hens in the same treatment group across all eight replicates were combined for statistical analysis. Tests for normality were conducted on all variables. In the case of the CORT data, Tukey’s boxplot method identified four outliers in CAF CO_2_ and one each from CAF and N_2_ treatment groups [34]. These data points were removed. Out of 185 samples used for the CORT assay, data of 179 samples were used in the statistical analysis. Statistical analyses of the data were conducted using a one-way analysis of variance following the PROC ANOVA procedure (SAS 9.4, Cary, NC, USA). Only main effects were considered in the statistical model as the test subjects differed only in terms of treatment applied. Means deemed significant were further analyzed using Fisher’s LSD test. All statistical tests were conducted at a 5% significance level. 

## 3. Results and Discussion

### 3.1. Foam Quality Parameters

**Figure 3:**
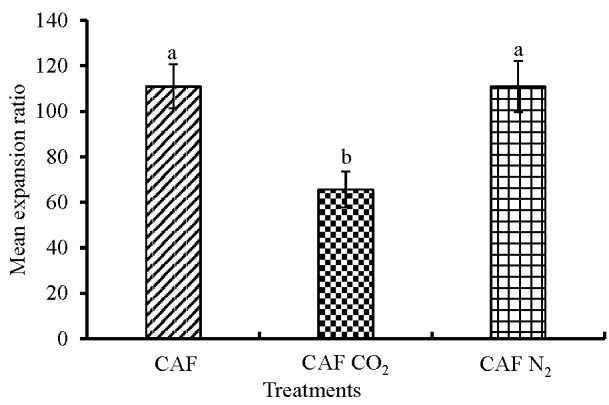
Mean expansion ratios of the three types of foam. The three foam treatments were CAF with air, CAF with CO_2_, and CAF with N_2_. The expansion ratio is the ratio of the volume of the finished foam to the volume of the aqueous foam solution. Bars (mean ± SEM) with different superscripts (a, b) are significantly different by Fisher’s LSD test (*p* < 0.05). The number of samples per treatment was 8.

Expansion ratios of all three kinds of foam and concentration of CO_2_ in the CAF CO_2_ foam were determined (Figure 3). The addition of CO_2_ in the foam, CAF CO_2_, significantly decreased the expansion ratio of the finished foam compared to CAF and CAF N_2_ (*p* = 0.004). The mean expansion ratios of CAF, CAF CO_2_, and CAF N_2_ were measured to be 111:1, 66:1, and 111:1, respectively. The average CO_2_ concentration achieved in the CAF CO_2_ foam across the eight replications was measured at 43%. However, the mass flow meter was set to obtain a 50/50 blend of CO_2_ and air. This discrepancy could be due to sample contamination and intact foam, which did not release enough gas for measurement from the headspace. 

Expansion ratio is one major factor affecting foam viscosity [35]. Low-expansion foams have lower viscosity [36] and, hence, they drain faster. In commercial layer operations, foam should be stable in cages for a considerable period to cause death of birds by mechanical hypoxia. The probable mechanism for a decrease in the expansion ratio of CAF CO_2_ foam was the reduction of pH of foam solution due to the formation of carbonic acid. Carbon dioxide gas reacting with water forms carbonic acid, H_2_CO_3_. Preliminary work on the measurement of the pH of compressed air foam with and without CO_2_ had determined the values to be 5.8 and 8.1, respectively. 

### 3.2. Serum Corticosterone

Corticosterone is the predominant glucocorticoid released from the adrenal cortex in rodents, birds, and reptiles [37]. Once released into the peripheral circulation, CORT binds to the intracellular glucocorticoid receptors. Glucose synthesis, lipolysis, and protein degradation are some of the effects of CORT used by an animal to cope with stressors [38].

**Figure 4:**
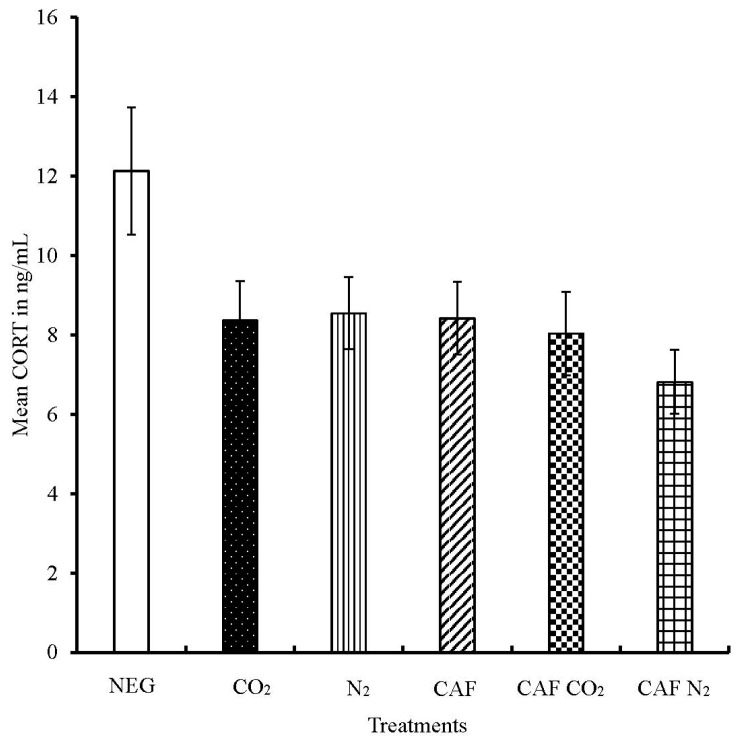
Mean serum corticosterone levels of spent hens. The CORT concentrations were measured in duplicates and expressed in ng/mL. Bars (mean ± SEM) with no superscripts are not significantly different by Fisher’s LSD test (*p* > 0.05). The total number of samples per treatment was 32.

The mean CORT levels of spent hens subjected to NEG, CO_2_, N_2_, CAF, CAF CO_2_, and CAF N_2_ treatments were 12.1 ng/mL, 8.4 ng/mL, 8.5 ng/mL, 8.4 ng/mL, 8.0 ng/mL, and 6.8 ng/mL, respectively (Figure 4). The CORT values of spent hens in all six treatment groups had no significant differences (*p* = 0.1249). However, the CORT level of spent hens subjected to the NEG group was numerically higher than the rest of the treatment groups. The CORT levels in all treatments, except the NEG group, indicate the endocrine response of the spent hens at or after their death. The spent hens in the NEG group were alive during the entire treatment period (4 min), unlike the foam and gas inhalation treatments, after which blood was collected by venipuncture. It is equally likely that these birds found the cage to be a novel environment than the floor pens where these birds were housed. All of these factors might have led to numerically elevated levels of CORT in hens in the NEG group. On the other hand, the spent hens in the CAF N_2_ group had the lowest CORT concentration among all six treatments, numerically. The three foam treatments CAF, CAF CO_2_, and CAF N_2_, did not differ (*p* > 0.05). The infusion of gases into CAF did not cause significant changes in the CORT concentration of spent hens as compared to the CAF treatment. The CORT concentration of spent hens subjected to foam treatments (CAF, CAF CO_2_, and CAF N_2_) did not differ significantly with that of the birds killed by the AVMA-approved poultry euthanasia method of gas inhalation, CO_2_ and N_2_ (*p* > 0.05). Similarly, Benson et al. [16] observed no significant differences in serum CORT levels of broilers among foam, foam with CO_2_, and CO_2_ polyethylene tent treatments. A previous study, in our lab, reported no significant differences in the serum CORT concentrations of young hens subjected to negative control, CO_2_ inhalation, and CAF treatments (paper currently under review).

### 3.3. Serum Serotonin

Serotonin, in birds, affects appetite, responses to fear, anxiety, and other stressors [39,40]. The serotonergic system in central nervous system has been demonstrated to be affected by handling and social separation in single combed White Leghorn chicks [41]. 

**Figure 5:**
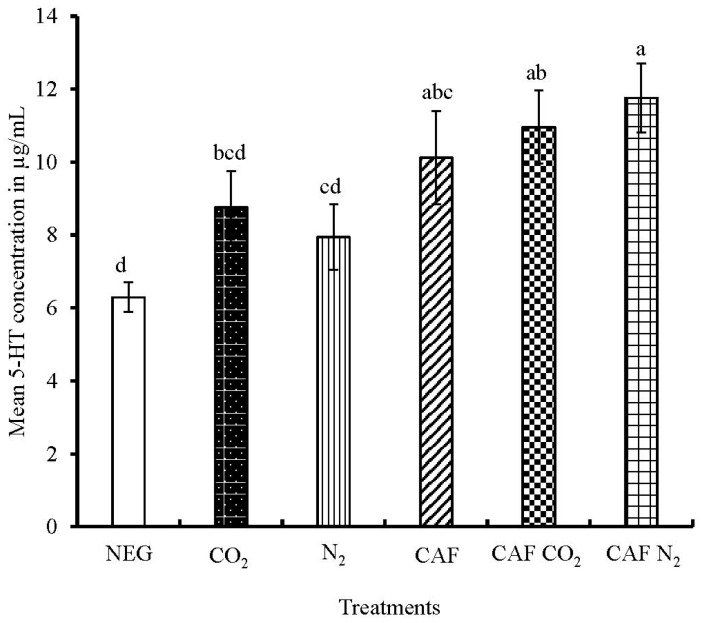
Mean serum serotonin levels of spent hens. The 5-HT concentrations were measured in duplicates and expressed in µg/mL. Bars (mean ± SEM) with different superscripts (a–d) are significantly different by Fisher’s LSD test (*p* < 0.05). The total number of samples per treatment was 16.

The mean serum 5-HT concentration of the hens in NEG, CO_2_, N_2_, CAF, CAF CO_2_, and CAF N_2_ were 6.3 µg/mL, 8.8 µg/mL, 7.9 µg/mL, 10.1 µg/mL, 11.0 µg/mL, and 11.7 µg/mL, respectively (Figure 5). The serum 5-HT levels of the spent hens differed significantly among the six treatments (*p* = 0.0010). The hens in the NEG group had significantly lower 5-HT levels as compared to CAF, CAF CO_2_, and CAF N_2_. The serum 5-HT levels of the hens in all treatments, except the NEG group, reflect the serotonergic response of the birds at or after death. The hens in the NEG group were alive longest for a period of four minutes after which blood was immediately collected. The cage might have also induced a fear response in these hens which led to a decrease in the serum 5-HT level. Bolhuis et al. [40] reported higher levels of whole blood 5-HT and less fear response in laying hens selected for low mortality due to feather pecking and cannibalism. However, foam treatments where gases were infused CAF CO_2_ and CAF N_2_ did not differ significantly with CAF in terms of mean 5-HT concentration. The 5-HT concentration of spent hens killed by the AVMA-approved euthanasia method of CO_2_ inhalation was similar to CAF and CAF CO_2_ treatments, but significantly lower than CAF N_2_ group. Birds in the N_2_ inhalation treatment had similar 5-HT levels to CAF, but were significantly lower than CAF CO_2_, and CAF N_2_ treatments. Higher levels of whole blood 5-HT was found to be associated with positive mood in human male volunteers [42], while higher concentration of corticosterone indicates higher stress levels [43]. These data may indicate that spent hens in the CAF N_2_ treatment had a lower anxiety and fear response as indicated by lower 5-HT levels than birds in the NEG, CO_2_, and N_2_ treatment groups. 

Uitdehaag et al. [44] suggested that peripheral 5-HT levels could be indicative of brain 5-HT activity in laying hens. Correlations between brain 5-HT and blood 5-HT were reported to be in the range from 0.34 to 0.57 [44]. Uitdehaag et al. [44] reported mean blood 5-HT levels of Rhode Island Red and White Leghorn hens in pure groups (birds of the same breed) after five minutes of manual restraint to be 11 µg/mL and 7.8 µg/mL, respectively. The 5-HT concentration of spent hens in our study varied from 6.5 µg/mL (NEG) to 11.7 µg/mL (CAF N_2_). In this study, spent hens in the NEG group had the highest CORT concentration (numerically), but the lowest 5-HT levels. In contrast, birds subjected to CAF N_2_ had the lowest CORT levels (numerically), but the highest 5-HT levels. Other studies have reported similar relationship between corticosteroids and brain 5-HT levels. Inoue and Koyama [45] observed that acute corticosterone administration decreased 5-HT in the hippocampus of rats. Similarly, Karten et al. [46] reported that chronic exposure to corticosteroid reduces 5-HT responses in hippocampus of rats. 

### 3.4. Time to Cessation of Movement

Animals subjected to euthanasia lose body posture, which is followed by the onset of clonic and tonic convulsions [47]. The cessation of convulsive movements is an indicator of brain death [48]. 

**Figure 6:**
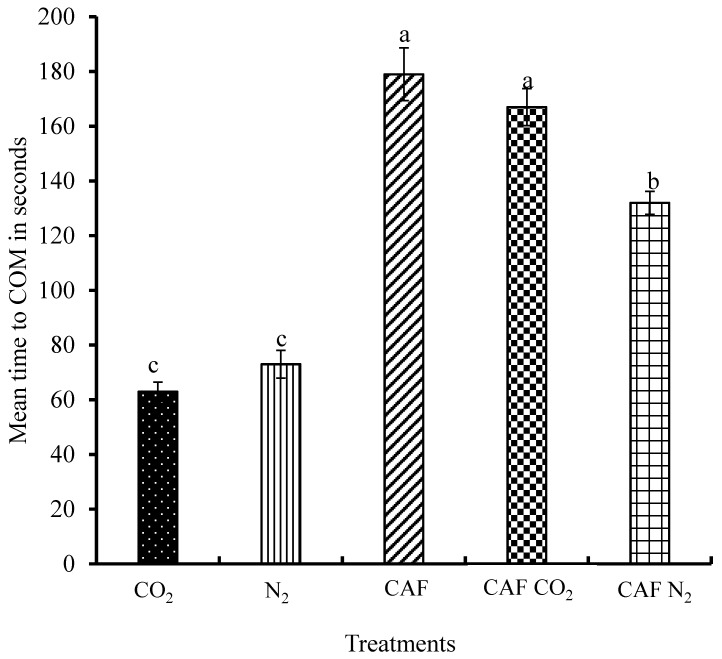
Mean time to cessation of movement of spent hens. The time was expressed in s. Bars (mean ± SEM) with different superscripts (a–c) are significantly different by Fisher’s LSD test (*p* < 0.05). The total number of samples per treatment was 32.

The times to COM of the spent hens to the five treatments (except NEG) were derived from the accelerometer readings logged from each hen. The spent hens in the CO_2_, N_2_, CAF, CAF CO_2_, and CAF N_2_ treatments took on average 63 s, 73 s, 180 s, 167 s, and 132 s to demonstrate COM, respectively (Figure 6). The time to COM differed significantly among the five treatment groups (*p* ≤ 0.0001). Spent hens exposed to the AVMA approved euthanasia methods of CO_2_ and N_2_ inhalation had significantly shorter time to COM than the birds exposed to rest of the treatments. These two methods resulted in faster death as indicated by the shortest time to COM. A previous study in our lab reported that spent hens subjected to CAF in cages took longer time to die than the hens exposed to CO_2_ in a chamber (paper under review). Birds subjected to CAF N_2_ treatment took significantly shorter time to the COM than the birds in the CAF and CAF CO_2_ treatments. Compressed air foam with N_2_ had better foam quality than CAF CO_2_. The foam bubbles contained N_2_ in CAF N_2_ while CAF had air. Therefore, the poor foam quality of CAF CO_2_ and the presence of air in the CAF might have led to delayed termination of convulsive movements in spent hens subjected to these treatments. 

The study was successful in developing compressed air foam infused with CO_2_ or N_2_. The data suggests that foam with N_2_ is advantageous than foam with CO_2_ by improving foam quality and reducing the time to death of caged laying hens during depopulation. Future studies should focus on replicating the process in a commercial layer facility. 

## References

[1] USDA APHIS (2016). Final Report for the 2014–2015 Outbreak of Highly Pathogenic Avian Influenza (HPAI) in the United States. https://www.aphis.usda.gov/animal_health/emergency_management/downloads/hpai/2015-hpai-final-report.pdf.

[2] Greene J.L. (2015). Update on the Highly-Pathogenic Avian Influenza Outbreak of 2014–2015.

[3] U.S. National List of Reportable Animal Diseases (NLRAD)-National Animal Health Reporting System (NAHRS) Operational Manual. https://www.aphis.usda.gov/animal_health/nahrs/downloads/nahrsoperationalmanual.pdf.

[4] Kinde H., Utterback W., Takeshita K., McFarland M. (2004). Survival of exotic Newcastle disease virus in commercial poultry environment following removal of infected chickens. Avian Dis..

[5] Thornton G. (2016). 9 Challenges Facing US Poultry Producers in 2017. http://www.wattagnet.com/blogs/6-all-things-poultry/post/29141-challenges-facing-us-poultry-producers-in-2017.

[6] AVMA Guidelines for the Euthanasia of Animals: 2013 Edition. https://www.avma.org/KB/Policies/Documents/euthanasia.pdf.

[7] USDA (2015). HPAI Outbreak 2014–2015 Stamping-Out & Depopulation Policy. https://www.aphis.usda.gov/animal_health/emergency_management/downloads/hpai/depopulationpolicy.pdf.

[8] USDA (2017). Highly Pathogenic Avian Influenza Response Plan: The RedBook. https://www.aphis.usda.gov/animal_health/emergency_management/downloads/hpai_response_plan.pdf.

[9] Hackbarth H., Küppers N., Bohnet W. (2000). Euthanasia of rats with carbon dioxide—Animal welfare aspects. Lab. Anim..

[10] Meyer R.E., Whitley J.T., Morrow W.E.M. (2013). Effect of physical and inhaled euthanasia methods on hormonal measures of stress in pigs. J. Swine Health Prod..

[11] Shields S.J., Raj A.B.M. (2010). A critical review of electrical water-bath stun systems for poultry slaughter and recent developments in alternative technologies. J. Appl. Anim. Welf. Sci..

[12] Otsuguro K., Yasutake S., Yamaji Y., Ban M., Ohta T., Ito S. Why does carbon dioxide produce analgesia?. Proceedings of the 6th World Congress on Alternatives & Animal Use in the Life Sciences.

[13] Martoft L., Lomholt L., Kolthoff C., Rodriguez B.E., Jensen E.W., Jørgensen P.F., Pedersen H.D., Forslid A. (2002). Effects of CO_2_ anaesthesia on central nervous system activity in swine. Lab. Anim..

[14] Kingston S.K., Dussault C.A., Zaidlicz R.S., Faltas N.H., Geib M.E., Taylor S., Holt T., Porter-Spalding B.A. (2005). Evaluation of two methods for mass euthanasia of poultry in disease outbreaks. J. Am. Vet. Med. Assoc..

[15] AVMA (2015). Poultry Depopulation. https://www.avma.org/KB/Policies/Pages/Poultry-Depopulation.aspx.

[16] Benson E., Malone G.W., Alphin R.L., Dawson M.D., Pope C.R., Van Wicklen G.L. (2007). Foam-based mass emergency depopulation of floor-reared meat-type poultry operations. Poult. Sci..

[17] Thornber P.M., Rubira R.J., Styles D.K. (2014). Humane killing of animals for disease control purposes. Rev. Off. Int. Epizoot..

[18] Gingerich E. Using Ventilation Shutdown for Emergency Mass Depopulation of Poultry. http://www.usaha.org/upload/Committee/TransDisPoultry/06-VSD%20for%20Mass%20Depopulation_Gingerich_share.pdf.

[19] McKeegan D.E.F., Reimert H.G.M., Hindle V.A., Boulcott P., Sparrey J.M., Wathes C.M., Demmers T.G.M., Gerritzen M.A. (2013). Physiological and behavioral responses of poultry exposed to gas-filled high expansion foam. Poult. Sci..

[20] Raj A.B. (1996). Aversive reactions of turkeys to argon, carbon dioxide and a mixture of carbon dioxide and argon. Vet. Rec..

[21] Sandilands V., Raj A.B.M., Baker L., Sparks N.H.C. (2011). Aversion of chickens to various lethal gas mixtures. Anim. Welf..

[22] Raj A.B.M. (2006). Recent developments in stunning and slaughter of poultry. Worlds Poult. Sci. J..

[23] Alphin R.L., Benson E.R., Hougentogler D.P., Herrman E.R. Is foam an option for addressing the challenges associated with the depopulation of caged layers?. Proceedings of the 5th International Symposium Managing Animal Mortalities, Products, By-Products, & Associated Heath Risks: Connecting Research, Regulations, & Responses.

[24] USDA (2016). HPAI Response Guidance: Using Ventilation Shutdown to Control HPAI. http://minnesotaturkey.com/wp-content/uploads/2015/03/USDA-NEW-Using-VSD-1.15.2016_V2.pdf.

[25] USDA (2015). HPAI Outbreak 2014–2015: Ventilation Shutdown Evidence & Policy. https://www.aphis.usda.gov/animal_health/emergency_management/downloads/hpai/ventilationshutdownpolicy.pdf.

[26] Zhang J.P., Delichatsios M., Neill A.O. (2011). Assessment of gas cooling capabilities of compressed air foam systems in fuel-and ventilation-controlled compartment fires. J. Fire Sci..

[27] Rie D.-H., Lee J.-W., Kim S. (2016). Class B fire-extinguishing performance evaluation of a compressed air foam system at different air-to-aqueous foam solution mixing ratios. Appl. Sci..

[28] Kim A.K., Dlugogorski B.Z. (1996). Multipurpose overhead compressed-air foam system and its fire suppression performance. J. Fire Prot. Eng..

[29] White D., Gurung S., Zhao D., Tabler T., McDaniel C., Styles D., McKenzie S., Farnell Y., Farnell M. (2017). Foam or spray application of agricultural chemicals to clean and disinfect layer cages. J. Appl. Poult. Res..

[30] Magrabi S.A., Dlugogorski B.Z., Jameson G.J. (2002). A comparative study of drainage characteristics in AFFF and FFFP compressed-air fire-fighting foams. Fire Saf. J..

[31] Farnell M., Caldwell D., Bryd A., Berghman L., Kiess A., Stayer P., Tabler T., Farnell Y. Use of a compressed air foam system in response to reportable poultry diseases. Proceedings of the 5th International Symposium Managing Animal Mortalities, Products, By-Products, & Associated Heath Risks: Connecting Research, Regulations, & Responses.

[32] Laundess A.J., Rayson M.S., Dlugogorski B.Z., Kennedy E.M. (2012). Suppression performance comparison for aspirated, compressed-air and in situ chemically generated class B foams. Fire Technol..

[33] Dawson M.D., Lombardi M.E., Benson E.R., Alphin R.L., Malone G.W. (2007). Using accelerometers to determine the cessation of activity of broilers. J. Appl. Poult. Res..

[34] Menon D.G., Bennett D.C., Schaefer A.M., Cheng K.M. (2013). Hematological and serum biochemical profile of farm emus (*Dromaius novaehollandiae*) at the onset of their breeding season. Poult. Sci..

[35] Gardiner B.S., Dlugogorski B.Z., Jameson G.J. (1998). Rheology of fire-fighting foams. Fire Saf. J..

[36] Dlugogorski B.Z., Kennedy E.M., Schaefer T.H., Vitali J.A. (2002). What Properties Matter in Fire-Fighting Foams?.

[37] Squires E.J. (2003). Applied Animal Endocrinology.

[38] Smith S.M., Vale W.W. (2006). The role of the hypothalamic-pituitary-adrenal axis in neuroendocrine responses to stress. Dialogues Clin. Neurosci..

[39] Metzger M., Toledo C., Braun K. (2002). Serotonergic innervation of the telencephalon in the domestic chick. Brain Res. Bull..

[40] Bolhuis J.E., Ellen E.D., Van Reenen C.G., De Groot J., Ten Napel J., Koopmanschap R.E., De Vries Reilingh G., Uitdehaag K.A., Kemp B., Rodenburg T.B. (2009). Effects of genetic group selection against mortality on behavior and peripheral serotonin in domestic laying hens with trimmed and intact beaks. Physiol. Behav..

[41] Gruss M., Braun K. (1996). Distinct activation of monoaminergic pathways in chick brain in relation to auditory imprinting and stressful situations: A microdialysis study. Neuroscience.

[42] Williams E., Stewart-Knox B., Helander A., McConville C., Bradbury I., Rowland I. (2006). Associations between whole-blood serotonin and subjective mood in healthy male volunteers. Biol. Psychol..

[43] Scanes C.G. (2016). Biology of stress in poultry with emphasis on glucocorticoids and the heterophil to lymphocyte ratio. Poult. Sci..

[44] Uitdehaag K.A., Rodenburg T.B., Van Reenen C.G., Koopmanschap R.E., De Vries Reilingh G., Engel B., Buist W.G., Komen H., Bolhuis J.E. (2011). Effects of genetic origin and social environment on behavioral response to manual restraint and monoamine functioning in laying hens. Poult. Sci..

[45] Inoue T., Koyama T. (1996). Effects of acute and chronic administration of high-dose corticosterone and dexamethasone on regional brain dopamine and serotonin metabolism in rats. Prog. Neuro-Psychopharmacol. Biol. Psychiatry.

[46] Karten Y.J., Nair S.M., van Essen L., Sibug R., Joëls M. (1999). Long-term exposure to high corticosterone levels attenuates serotonin responses in rat hippocampal CA1 neurons. Proc. Natl. Acad. Sci. USA.

[47] Gerritzen M., Lambooij B., Reimert H., Stegeman A., Spruijt B. (2007). A note on behaviour of poultry exposed to increasing carbon dioxide concentrations. Appl. Anim. Behav. Sci..

[48] Erasmus M.A., Turner P.V., Widowski T.M. (2010). Measures of insensibility used to determine effective stunning and killing of poultry. J. Appl. Poult. Res..

